# Downregulation of HMGA2 by Small Interfering RNA Affects the Survival, Migration, and Apoptosis of Prostate Cancer Cell Line

**DOI:** 10.34172/apb.2022.039

**Published:** 2021-04-03

**Authors:** Shima Khajouee, Elham Baghbani, Ali Mohammadi, Behzad Mansoori, Dariush Shanehbandi, Khalil Hajiasgharzadeh, Behzad Baradaran

**Affiliations:** ^1^Immunology Research Center, Tabriz University of Medical Sciences, Tabriz, Iran.; ^2^Connective Tissue Diseases Research Center, Tabriz University of Medical Sciences, Tabriz, Iran.; ^3^Pharmaceutical Analysis Research Center, Tabriz University of Medical Sciences, Tabriz, Iran.; ^4^Neurosciences Research Center, Tabriz University of Medical Sciences, Tabriz, Iran.

**Keywords:** HMGA2, Gene therapy, Small interfering RNA, Prostate cancer, Apoptosis assay, Cell migration

## Abstract

***Purpose:*** To investigate the downregulation of high mobility group AT-hook 2 (HMGA2)expression by small interfering RNAs (siRNAs) in PC3 prostate cancer cell line. HMGA2belongs to the non-histone chromatin-binding protein family that serves as a crucial regulator ofgene transcription. The overexpression of this gene is positively correlated with various prostatecancer (PC)-related properties. Thus, HMGA2 is an emerging target in PC treatment. This studyaimed to examine the impact of siRNAs targeting HMGA2 on the viability, migration, andapoptosis processes of the PC3 PC cell line.

***Methods:*** siRNA transfection was conducted with a liposome-mediated approach. The mRNAand protein expression levels for HMGA2 are evaluated by real-time polymerase chain reaction(qRT-PCR) and western blot analysis. The cytotoxic properties of HMGA2-siRNA were measuredby MTT assay on PC3 cells. The migration of PC3 cells was measured by implementing awound-healing assay. Apoptosis measurement was also quantified by TUNEL assay.

***Results:*** Transfection with siRNA significantly decreased both mRNA and protein levels of theHMGA2 gene in a dose-dependent manner after 48 hours. Also, we demonstrated that theknockdown of HMGA2 led to a reduction in cell viability, migration ability, and enhancedapoptosis of PC3 cells in vitro.

***Conclusion:*** Our findings recommend that the specific siRNA of HMGA2 may efficiently beable to decrease PC progression. Therefore, it may be a promising adjuvant treatment in PC.

## Introduction

Prostate cancer (PC) is the most prevalent non-dermatologic cancer in males.^1^ Moreover, it is the second major cause of cancer-associated death in the male population in most western societies.^2^ Epidemiologic studies revealed that PC incidence and its mortality rates are remarkably different among the populations. The PC rate is low in Asia, intermediate in Eastern Europe and Africa, and the highest rates are in North America and Western Europe.^3^ High mobility group AT-hook 2 (HMGA2) protein (formerly HMGI-C), as a non-histone chromatin-binding protein, belongs to a family of small high-mobility-group (HMG). It contains three AT-hook parts that facilitate the binding process of the protein B-form DNA in a specific A-T rich region.^4,5^ This protein is highly expressed in embryonic stem cells during embryonic development and in the fetal period, but it is normally expressed at low levels in adult tissues and differentiated cells.^6^ Furthermore, HMGA2 is frequently found to be upregulated in cancers of mesenchymal origin and metastatic cancers, implying that it may have a pivotal role in controlling cell proliferation.^7,8^ HMGA proteins are crucial for a variety range of cellular functions, such as gene transcription, cell division, mitosis, cell cycle progression, cancer initiation, metastasis, cellular aging, and differentiation.^9-11^

In humans, overexpression of HMGA2 gene was detected in different tumors such as leukemia,^12,13^ epithelial ovarian cancers,^14^ myeloproliferative disorders,^15^ uterine tumorigeneses,^16^ lymphoid malignancy,^17^ pancreases,^18^ retinoblastomas,^19^ non-small cell lung^20^ and oral squamous cell,^21^ which is an indicator of poor prognosis. Moreover, HMGA2 is involved in the initiation of epithelial-to-mesenchymal transition (EMT) in a human PC cell line (PC-3).^22^ These experiments recommend that HMGA2 has an important impact on various tumors, such as PC, which is supported by the re-expression of HMGA2 as a prognostic tumor marker. Considering these findings, in this study, we determined the anti-cancer influences of small interfering RNA (siRNA) on the PC3 cell line by targeting HMGA2.

## Materials and Methods

### Main materials

The siRNA sequence against HMGI-C, anti-HMGI-C and b-actin antibodies, RIPA lysis reagent, and siRNA transfection reagent and medium were bought from Santa Cruz Biotechnology (CA, USA). Secondary antibodies were bought from the Cytomatin gene company (Isfahan, Iran).

### Cell culture

The human prostate cancer cell line (PC3) was obtained from the Pasteur Institute of Iran. PC3 cells were cultivated in RPMI-1640 medium that was completed with 10% FBS and penicillin/streptomycin mixtures (Gibco, USA) under standard cell culture conditions. The cells were then used according to our previous experiments.^23^

### siRNA transfection

PC3 cells were cultured at 2×10^5^ cells/well in 6-well plates and grown to 80% confluency. The siRNA at a final dose of 80 nM, using a siRNA transfection reagent, was transfected into the cells. In brief, siRNA and its transfection reagents diluted in the siRNA transfection medium in another tube. Following, the diluted siRNA and transfection reagent were mixed in other tubes and kept for about half-hour at room temperature (RT). Subsequently, the mixed solution was added into the well and the cells culture incubated for 6 hours. Ultimately, the siRNAs in the Opti-MEM solution were applied to the cells. After 48 hours of siRNA transfection, the effects of siRNA was assessed by applying quantitative real-timepolymerase chain reaction (qRT-PCR) and western blotting to approve transfection efficacy.^24^

### Quantitative real-time PCR (qRT-PCR)

Total RNA was isolated from the cells by RNX-plus reagent (Bioneer, Korea).

The cDNA was synthesized from 1μg of total RNA and the qRT-PCR was done using the Rotor-Gene system (Corbett Life Science, Australia) and reported by the 2^−ΔΔCT^ method. The sequences of the primers were represented in Table 1. β-actin was monitored as the reference gene. All qRT-PCR reactions were performed in triplicate.

**Table 1:** Primer sequences in real-time PCR

**Target gene**	**Strand**	**Primer sequence**
β actin	Forward	5’-TCCCTGGAGAAGAGCTACG-3’
	Reverse	5’-GTAGTTTCGTGGATGCCACA-3’
HMGA2	Forward	5’-TGGGAGGAGCGAAATCTAAA-3’
	Reverse	5’-TGGTATTCAGGTCTTTCATGG-3’

### Western blot analysis

Total proteins were extracted from PC3 cells by RIPA buffer. Shortly, 100 µL of lysis buffer, protease inhibitors, and PMSF, were gathered and kept on ice for fifteen min. After that, the proteins were precipitated following five min centrifugation at 14 000 g in 4°C. A NanoDrop spectrophotometer (Thermo Scientific, USA) evaluated the purity of extracted proteins. The protein was electrophoresed with SDS–PAGE and then transferred to a PVDF membrane (Roche, Basel, Switzerland). After transferring, the membranes were blocked with 0.5% tween 20 in PBS/Tween-20 (0.05%, v/v). After that, the membranes were incubated for 1 hour at RT with primary HMGA2 goat antibody (1:2000) and β-actin (1:5000), which were diluted in 3% BSA in PBS. Following 4-time washing, the membranes were probed with secondary antibodies (1:5000) for 1 hour on a shaker at RT. Then, the protein bands were detected by the electrochemiluminescence detection kit (Roche Diagnostics GmbH, Mannheim, Germany). β-Actin was used to verify the loading of the same amount of proteins. The signals were measured via ImageJ Software.

### MTT assay

The cytotoxicity assay was done by using MTT colorimetric method. The study was divided into six groups: 80 nM HMGA2-siRNA, 60 nM HMGA2-siRNA, 40 nM HMGA2-siRNA, Transfection Reagent, Pure siRNA and, control group. Briefly, 15×10^3^ cells were seeded in 96-well plates. After transfection, 50 µL of MTT (2 mg/mL in PBS) was incorporated into the wells and kept for an additional 4 hours. Then, the medium was discarded by the addition of 200 µL of DMSO and Sorensen buffer mixtures. The formazan crystals were produced, and after 30 min of incubation in the same condition. Finally, the optical density indices were measured with an ELISA Reader at 570 nm.

### Migration assay

The migration of PC3 cells was assessed by implementing a wound-healing assay (Scratch). PC3 cells (4×10^5^ cells/well) were cultivated into 6-well plates for 24 hours and when the cells reached >90 % confluency, a wound was produced by dragging a yellow pipette tip across the PC3 cells layer. Subsequently, the photographs of the cells were taken by an inverted microscope (Optika, XDS-3, Italy) at 0, 24, and 48 hours. Images of the wound area were obtained at 0, 24, and 48 hours by a light microscope. The migratory ability of the cells was assessed by the measurement of the gap between the wound edges.

### Apoptosis assay

To estimate the apoptosis of the PC3 cells, the TUNEL assay was done following the instructions. Briefly, cells were seeded in 96-well plates (15×10^3^ cells/well) and transfected as explained above. Afterward, cells were fixed with 4% paraformaldehyde solution (Sigma Aldrich, USA) for 48 hours at RT. Then, cells were incubated with 0.3% H2O2-CH3OH mixture and Triton X-100 for 2 minutes on ice.

### Statistical analysis

The results are described as mean ± standard deviation (SD). Statistical comparisons among the groups were done, by using one-way ANOVA followed by Dennett’s multiple comparisons through GraphPad Prism software. The value of *P* < 0.05 was regarded as a significant difference.

## Results and Discussion

After the introduction of the RNA interference (RNAi) technology a few years ago, the potential of using the gene silencing approach generated an excellent deal of interest. Identifying genes that are commonly expressed in cancer cells but not in healthy cells is a critical issue. HMGA2 appears to be such a candidate. HMGA2 is a nuclear architectural protein, which binds to DNA sequences to regulate the transcription activity by modulating chromatin structure. Besides, high levels of HMGA2 have been recognized in numerous benign and malignant cancers. HMGA2 is an important stem cell factor that is overexpressed in undifferentiated cells in fetal development but silenced in adult human tissues. Furthermore, the up-regulation of HMGA2 has been connected to malignant phenotypes such as cancer proliferation, chemoresistance, enhanced metastasis, and poor prognosis in numerous kinds of malignancies.^25-29^ In this present report, we investigated the expression level of HMGA2 on the PC3 cell line to determine its possible role as a prognostic biomarker.

### siRNA against HMGA2 represses the mRNA and protein expression of PC3 cells

**Figure 1:**
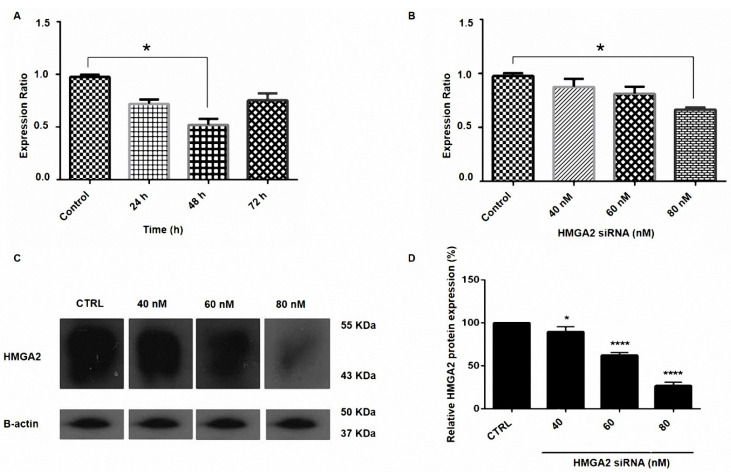
Suppression of HMGA2 expression by siRNA in prostate cancer. (A) PC3 cells were transfected with HMGA2 siRNA for 24, 48, and 72 h. (B) PC3 cells were transfected with HMGA2 siRNA with the doses of 40, 60, 80 nM. Relative HMGA2 mRNA expression was measured by qRT-PCR using the 2^-ΔΔCt^ method (*P* < 0.05). (C) The expression of HMGA2 protein in PC3 cells transfected with siRNA. Representative western blot of beta-actin and HMGA2 proteins from cells transfected with HMGA2 siRNA. (D) Results indicated the reduction in the transfected cells in 80 nM dose of siRNA and the expression level of each band was quantified using densitometry and normalized to the respective beta-actin. *P < 0.05 and ****P < 0.0001, versus control.

PC3 cells were transfected with HMGA2-siRNA and then the effect of siRNA on the expression of HMGA2 was detected by qRT-PCR and western blotting methods. The β-actin gene expression served as a control group. As exhibited in Figures 1A and 1B, the siRNA of HMGA2 led to a meaningful reduction in the mRNA levels of HMGI-C mRNA (*P* < 0.05). The results from the western blot tests designated that HMGA2 was efficiently repressed in PC3 cells (Figure 1C and 1D).

### HMGA2 suppression caused cytotoxic PC3 cells in a dose-dependent manner

**Figure 2:**
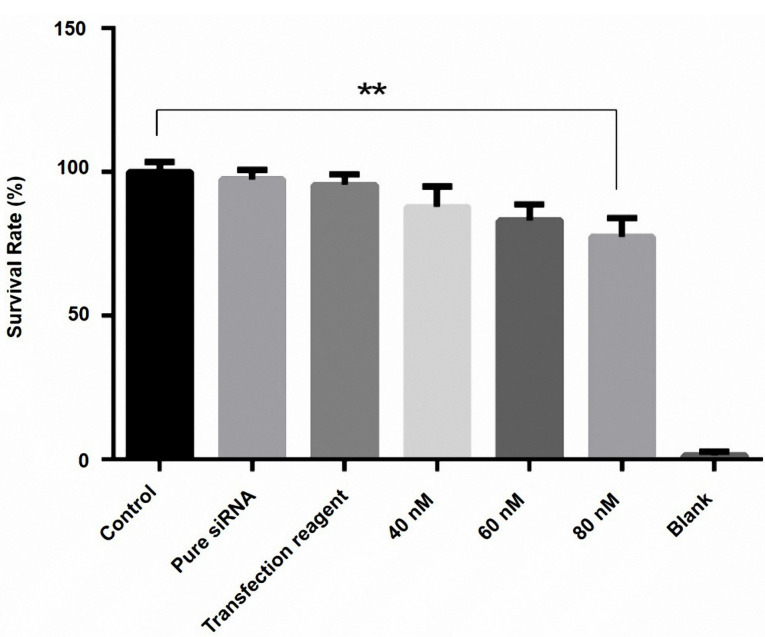
Effect of HMGA2 siRNA on cell survival in the PC3 cell line. Cell survival was assayed by MTT assay 48 h after transfection of three doses of HMGA2 siRNA (40, 60, and 80 nM), as described in the methods. At 48 h after transfection with (40, 60, and 80 nM), the viability of cells was assessed by MTT assay. The results showed that HMGA2 siRNA reduced the cell survival rate in a dose-dependent manner. Among these different doses, the transfection of 80 nM HMGA2-siRNA showed the greatest decrease in survival rate. **P < 0.05 versus control.

The viability of the cells was monitored by MTT assay. The findings indicated that HMGA2 siRNA reduced the cell survival rate in comparison to controls (*P* < 0.05). Furthermore, we have identified that the transfection of 80 nM HMGA2-siRNA reduced the rate of PC3 cell viability to 23% in comparison to control cells (*P* = 0.0003). Also, the transfected groups with transfection reagent and pure siRNA did not display a significant difference with control cells (Figure 2).

### Downregulation of HMGA2 reduces the cells migration ability

**Figure 3:**
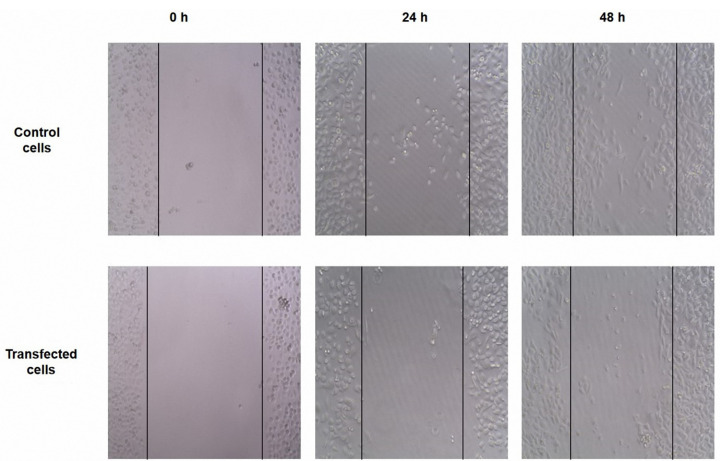
Untreated and treated PC3 cells were subjected to scratch wound-healing assays. The wound space was photographed at 0, 24, and 48 h. The results showed that the transfection of PC3 cells with HMGA2-siRNA caused the reduction of the cell migration in after 24 h, in comparison to the migration rate of the control cells.

The effects of HMGA2 was verified in the cell migration by knockdown of HMGA2 through siRNA in PC3 cells. It is shown that it would lead to the blocking of cell migration ability. The wound healing approach was done to assess whether the inhibitory influence of cell migration of the PC3 cells that transfected with HMGA2-siRNA is mediated through its inhibitory effect on HMGA2 expression. The results showed that the transfection of PC3 cells with HMGA2-siRNA caused the reduction of the cell migration in PC3 cells after 24 hours, in comparison to the migration rate of the control cells (Figure 3).

### Downregulation of HMGA2 enhanced apoptosis in PC3 cells

**Figure 4:**
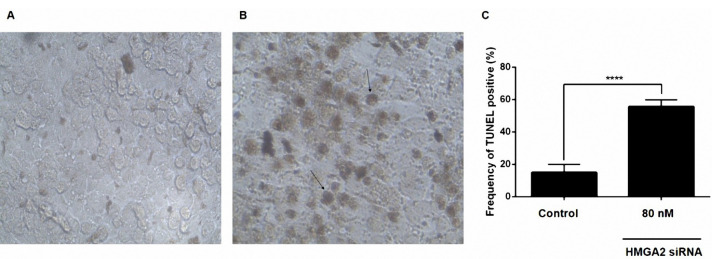
siRNA-mediated targeting of HMGA2 significantly sensitized PC3 cell lines by stimulation of apoptosis measured by in situ cell death detection kit. (A) Untreated siRNA as a negative control. (B) PC3 cell lines treated with 80 nM HMGA2-siRNA. (C) Percentage of TUNEL-positive cells in the control and test groups. Transfecting the PC3 cells with HMGA2-siRNA resulted in a reduced number of living cells and increased the frequency of TUNEL positive cells after 48 h and at the concentration of 80 nM of HMGA2-siRNA in comparison to the non-transfected cells. ****P < 0.0001 versus control.

The effects of the downregulation of HMGA2 on apoptosis of PC3 cells were determined by the TUNEL assay. Transfecting the PC3 cells with HMGA2-siRNA resulted in a reduced number of living cells after 48 hours and at the concentration of 80 nM of HMGA2-siRNA in comparison to the non-transfected cells (Figure 4 A-C).

In line with our findings, the association between cancer proliferation and the expression level of HMGA2 has been reported, previously. It was shown that the knockdown of HMGA2 reduced the growth of the tumor cells. We observed that the expression of HMGA2 in the PC3 cell line was increased. Malek et al showed that about 65 percent of ovarian carcinoma cells are positive for HMGA2. But in this study, all healthy tissues were negative for the expression of this protein.^14^ Shell et al investigated HMGA2 expression by immunohistochemistry and founded that the overexpression of HMGA2 is associated with a poor prognosis.^30^ In another similar study, Low et al indicated that the inhibition of BIRC6 by siRNAs led to significant suppression of PC cell viability.^31^ Sun et al showed that HMGA2 was correlated with metastasis and diminished prognosis in gastric cancer, and a xenograft mouse model and overexpression of the HMGA2 gene promoted tumor growth.^32^ Shi et al revealed that knockdown of HMGA2 effectively suppressed the migration, proliferation, invasion, and also promoted the apoptosis rate of PC cells.^22^ In our study, we designed the experiments in a step-by-step manner. First, we obtained the optimal dose and time of siRNA transfection. Then, the efficacy of the siRNA proved in reducing gene expression in both mRNA and protein levels. We demonstrated that the HMGA2 specific siRNA efficiently reduces PC cell viability and induces apoptosis. In our study, the reduced protein level of HMGA2 after transfection was identified by western blotting. Also, the cytotoxicity and apoptosis were measured by MTT and TUNEL assays, respectively. Besides, the scratch wound healing technique was applied to evaluate the impact of HMGA2 expression on the migration of the cells. The findings showed the inhibitory effects of its knockdown on the migration of PC cells.

## Conclusion

In the current study, we designated that the knockdown of HMGA2 can markedly inhibit cell proliferation, which is related to the high apoptosis rate. Furthermore, our results confirmed that the silencing of HMGA2 in the cancer cells considerably inhibited cell migration. Therefore, it can be considered as a potent adjuvant in PC therapy.

## Acknowledgments

This work was financially supported by grant from Tabriz University of Medical Sciences, Tabriz, Iran (grant number 63785). The authors would like to thank them for their support.

## Ethical Issues

All experiments and procedures were conducted in compliance with the ethical principles of Tabriz University of Medical Science, Tabriz, Iran and approved by the regional ethical committee for medical research (Ethical code: IR.TBZMED.REC. 1398.321).

## Conflict of Interest

The authors declare that they have no conflict of interest.
